# Brothers and sisters sharing in the care of a parent with dementia

**DOI:** 10.1177/14713012211053970

**Published:** 2022-02-03

**Authors:** Kristina M Kokorelias, Nira Rittenberg, Amy Law, Natasha T Chin Wan, Jennifer Machon, Yasmin Arfeen, Jill I Cameron

**Affiliations:** Rehabilitation Sciences Institute, Temerty Faculty of Medicine, University of Toronto, St. John’s Rehab, 71545 Research Program, Sunnybrook Research Institute, Toronto, ON, Canada; Faculty of Medicine to Temerty Faculty of Medicine, 7938 University of Toronto, Toronto, ON, Canada; Department of Occupational Science and Occupational Therapy, Temerty 12366 Faculty of Medicine, University of Toronto, Toronto, ON, Canada; Department of Occupational Science and Occupational Therapy, Temerty 12366 Faculty of Medicine, University of Toronto, Toronto, ON, Canada; Department of Occupational Science and Occupational Therapy, Temerty 12366 Faculty of Medicine, University of Toronto, Toronto, ON, Canada; Department of Occupational Science and Occupational Therapy, Temerty 12366 Faculty of Medicine, University of Toronto, Toronto, ON, Canada; Department of Occupational Science and Occupational Therapy, Rehabilitation Sciences Institute, Temerty 12366 Faculty of Medicine, University of Toronto, Toronto, ON, Canada

**Keywords:** brother, sister, dementia, siblings, parent, caregiver, relationship

## Abstract

Brothers’ and sisters’ experiences providing care to a parent with dementia differ, but little is known about how mixed-gender siblings share their caregiving responsibilities or how sharing affects their relationship. This study aimed to explore mixed-gender siblings processes for distributing caregiving tasks when caring for a parent with dementia and the impact of sharing care on their relationship. This descriptive qualitative study recruited fourteen English-speaking mixed-gender sibling pairs caring for a parent with dementia. Online open-ended surveys and individual semi-structured interviews were completed. Interviews and surveys explored division of caregiving responsibilities, conflict resolution, and the effects of sharing care on sibling relationships. Thematic analysis was used to analyze the qualitative data. Five themes were identified: goal of shared caregiving is to meet parents’ needs, sisters often take the lead, practical issues affect sharing of caregiving activities, personal resources or skills affect division of responsibilities, and shared caregiving influences relationship quality. Understanding how siblings share caregiving responsibilities can inform the practices of healthcare professionals who care for people with dementia and their family caregivers.

## Introduction

Dementia is broadly defined as a set of symptoms caused by disorders of the brain. The symptoms of dementia include loss of memory, difficulties with language, and deficits in the thinking and problem-solving capacity of an individual that are so acute that they diminish an individual’s ability to perform activities of daily living (Alzheimer’s Society of Canada, 2019). Spouses tend to be the initial primary caregivers for people with dementia and are heavily involved in providing care (Pinquart & Sörensen, 2011). As they age and face declines in their own health, their children become increasingly involved in caregiving. Caregiving that is shared between family members can often be unequal and this can impact their relationship quality (Barber and Pasley, 1995; Broadty and Donkin, 2009; Panyavin et al., 2015).

Gender-differences in the time spent on caregiving tasks have been noted. Consistent across the literature, women contribute more hours and types of care to persons with dementia than men (Coward & Dwyer, 1990; Chang & White-Means, 1991; Pillemer et al., 2018; Revenson et al., 2016), regardless of external factors such as time demands, attitudes toward obligation, and available resources (Erol, Brooker, & Peel, 2016; Gerstel & Sarkisian, 2015). Although women have typically assumed the caregiver role, the proportion of male caregivers is increasing (Hellström, Håkanson, Eriksson, & Sandberg, 2017)

There are conflicting findings as to whether the experience of dementia caregiving differs among male and female adult children caregivers in terms of types of caregiving tasks provided. Brothers are more likely to help with tasks such as finances, house repairs, yard work, and transportation (Lai, Luk, & Andruske, 2007; Stoller, 1994) and typically perform more of a care manager role (e.g., management and practical supports) (Grigorovich et al., 2016). Other studies posit that daughters are more likely to assist with instrumental activities (including meal preparation and household chores), personal care, and emotional support (Connidis & Barnett, 2018; Grigorovich et al., 2016; Horowitz, 1985). Many studies examining adult–child caregivers’ perspectives on their shared caregiving role, its impact on other aspects of life, and the dynamic with their siblings focused solely on the perspectives of one sibling (Kramer and Kipnis, 1995; Barber and Pasley, 1995; Ingersoll-Dayton et al., 2003a; Ingersoll-Dayton et al., 2003b). Therefore, more research is required to examine the perspectives of both the sons and daughters who share dementia care responsibilities for the same parent. Research findings also highlight the process of sharing care tasks amongst sibling who provide care to an elderly parent. In an effort to equally distribute caregiving responsibilities, siblings may take turns to complete tasks and divide tasks based on their expertise (Connidis & Barnett, 2018; Ingersoll-Dayton et al., 2003a). Additionally, siblings may address inequality by asking siblings to increase their level of involvement, such as more visits with parents or greater financial assistance (Ingersoll-Dayton et al., 2003b). Siblings who contribute more may be in distress and feel resentful towards their other siblings (Ingersoll-Dayton et al., 2003b). When the distribution of tasks is not equitable and interferes with work, family obligations, or leisure, caregivers report more burden (Kramer and Kipnis, 1995; Ingersoll-Dayton et al., 2003b). This can ultimately affect parent care. As brothers are increasingly assuming the caregiver role, their experiences in relation to sharing care with their sisters is worthy of additional research (Pillemer et al., 2018).

Brothers’ and sisters’ experiences providing care to a parent with dementia differ but little is known about how mixed-gender siblings share their caregiving responsibilities or how sharing affects their relationship. The objective of this qualitative descriptive study was to explore the relationship between mixed-gender siblings and their processes for distributing caregiving tasks when providing care for a parent with dementia residing at home. The research questions guiding this study were: (1) What are mixed-gender sibling caregivers’ perspectives regarding shared responsibilities in their parent’s dementia care? (2) How do mixed-gender sibling caregivers share caregiving responsibilities? and (3) How does sharing care responsibilities impact relationship between siblings?

## Design and methods

### Design

A qualitative descriptive research design (Sandelowski, 2000; 2010) was used to explore siblings’ perspectives regarding caregiving roles and how caregiving tasks are shared.

This study collected data in two phases. The first phase involved an online open-ended survey. This aim of the first phase was to gain initial insight into how siblings described their caregiving roles and the sharing of tasks. These preliminary insights informed development of our semi-structured interview guide used during the second phase. The second phase involved qualitative semi-structured telephone interviews. The interviews facilitated probing participants’ caregiving experiences in more depth. A thematic analysis was conducted with phase one and two data to identify the key themes. This study received approval from the Research Ethics Board at The University of Toronto.

## Methods

### Participants

Fourteen mixed-gender sibling pairs were recruited and completed data collection. The participants met all of the following inclusion criteria: (1) a son and daughter pair; (2) 18 years of age or older; (3) able to speak and understand English; (4) residing in Canada; and (5) caring for a parent with a clinical diagnosis of dementia. Individuals whose parent resided in a long-term care facility or nursing home for longer than 6 months were excluded as our aim was to understand shared caregiving in the context of home care. Purposive sampling was used to target the specific population needed for this study (i.e., mixed-gender sibling pairs) and convenience sampling for its timeliness, ease of use, accessibility, availability, and affordability (Merriam & Tisdell, 2016). Participants were recruited through social media (e.g., Twitter and Facebook) and from community organizations (e.g., Alzheimer’s Society and their chapters) to maximize exposure to this target population.

### Data collection

Data were collected from 2016 to 2019. Online surveys were initially used to increase accessibility. We adapted the online survey for use in a semi-structured interview (see Supplemental Material 1). Four participant-dyads (brother–sister pairs) completed the online surveys (8 surveys). Ten dyads participated in the qualitative semi-structured interviews (20 interviews total). Participants who were interviewed did not complete the survey. Each sibling was interviewed separately to allow them to speak freely about their experiences. Four members of the research team conducted the interviews (AL, NT, JM, and YA). The interviewers were Masters of Science in Occupational Therapy students, trained in qualitative research methods. The semi-structured interviews discussed the following topics: (1) quality of relationship with siblings; (2) how caregiving roles are shared; and (3) type of care provided by each sibling. Sociodemographic information, such as age, marital status, employment status, and number of siblings, was collected to characterize the sample. Interviews were audio recorded, transcribed verbatim, and reviewed for accuracy.

### Data analysis

Data analysis was led by KMK, NR, and JIC. Data were analyzed using the thematic analysis guidelines set by Braun and Clarke (2006). KMK, AL, NTCN, JM, and YZ coded the data by first becoming familiar with the data (reviewing the transcripts and/or audio files numerous times) and generating initial codes based on the research questions. This list of codes was reviewed by KMK, NR, and JIC who provided feedback and determined an exhaustive coding scheme that was then applied to all the transcripts by KMK, AL, NTCN, JM, and YZ. Any additional novel codes that were not captured in the original coding scheme were discussed among the research team and applied to the transcripts. Analyses then included the research team comparing and contrasting the coded data to categorize similar ideas. KMK, NR, and JIC then reviewed the raw and coded data to develop initial themes. All authors contributed to clarifying and refining the themes generated. Once saturation of themes was established, the themes were given a title that reflected the data.

Investigator triangulation and an audit trail of the study process were used to ensure reliability of the interpretation of the data (Merriam & Tisdell, 2016).

## Findings

Fourteen pairs of siblings participated in the study. Seven pairs provided care to their father, and seven pairs provided care to their mother. See Table 1 for participant characteristics. Online survey data included 53 pages of single-spaced text. Phone interviews ranged from 44 min to 77 min, with a median of 65 min. Our analysis revealed one overarching theme: goal of shared caregiving is to meet parents’ needs and four main themes that describe how caregiving is shared and how shared caregiving impacts sibling relationships. These four themes are as follows: (1) sisters often take the lead, (2) practical issues affect sharing of caregiving activities, (3) personal resources or skills affect division of responsibilities, and (4) shared caregiving influences relationship quality. The following is a summary of the themes with participant quotations as examples. The source of each quotation is indicated by sibling pair (SP), pair number, followed by sister or brother.

**Table 1.** Participant data (*N* = 14).

Characteristic	Median (range) or frequency
Daughter caregivers	
Age, years	54.5 (32–67)
Relationship status	—
Married or common law	4
Single or divorced	3
Employment status	—
Working for pay	8
Not working (retired, student, on disability)	1
Length of time caregiving	3.5 (0.5–10)
Amount of time for caregiving	10 (6–20)
Number of siblings	2 (1–6)
Son caregivers	
Age, years	55 (40–62)
Relationship status	—
Married or common law	5
Single or divorced	2
Employment status	—
Working for pay	7
Not working (retired, student, on disability)	0
Length of time caregiving, years	3 (0.5–10)
Amount of time for caregiving	7 (4–12)
Number of siblings	2 (1–6)
Care recipients	
Father	7
Mother	7
Stage of dementia^a^	—
Mild	3
Moderate	10
Severe	1
Living situation	—
In own home with spouse/family	8
In own home	3
With another caregiver	3

^a^Based on participants’ description of their parent’s need for assistance: *mild =* displaying a few symptoms, requiring little or no care; *moderate =* displaying multiple symptoms, requiring a greater level of care; and *severe =* displaying multiple symptoms, requiring full-time assistance.

### Overarching theme: Goal of shared caregiving is to meet parents’ needs

An overarching theme emerged from our study—the goal of shared caregiving is to meet parents’ needs—both the parent with dementia and, if available, their spousal, partner caregiver. In order to meet their parents’ needs, the mixed-gender siblings shared the belief that frequent and open communication was critical to keeping their parent’s best interests at the forefront. Examples of meeting the needs of a parent may include running errands, attending medical appointments, and arranging services.

The majority of the participants expressed satisfaction with the way care tasks are divided and not many conflicts arose between the pairs. Participants reported that this is likely due to the fact that they have a common goal of wanting to meet the person with dementia’s needs. As one participant expressed:

“I think all of us realize we’re all there to help my mother (person with dementia), but I think that helps a lot that nobody really feels like people are trying to shirk responsibilities. I think as a family we’re fantastic in a crisis. When a crisis happens, everybody jumps, everybody shares, everybody is willing to help” (SP5 Sister).

Siblings often shared the desire to keep their parent residing at home for as long as possible as they felt it aligned with their desires. However, the siblings unanimously agreed that as their parent’s abilities deteriorated and as the caregiver burden heightened, it would no longer be feasible for their parent to remain at home. They believed that after a certain extent of disease progression, it would become appropriate to for them to transition the person with dementia into a long-term care home. Sibling caregivers also tried to include their parent in the decision-making as much as possible in the early stages of their disease. However, as the disease progressed and their parent experienced more cognitive challenges, participants placed less emphasis on this. Siblings no longer felt confident that their parent could make informed decisions regarding their own care and believed they could more feasibly act in their parent’s best interests.

Some participants explained that it was also important to support both parents not just the parent with dementia. For example, “My father (parent without dementia) needed help and I stepped in and began to take my mom out of the house and cook, do laundry, help with wardrobe and hygiene and help with any parts of her care” (SP3 Sister). Her brother expressed, “My sister and I book time to assist in order to give our father a break and spend more time with our mother. We also cook food for them and help with chores when required” (SP3 Brother).

Meeting the parents’ care needs was not always related to functional tasks and would often be in the form of keeping their parents’ company and spending quality time with one another. For example, SP9 (Brother) expressed that a large portion of their time is dedicated to “spending time with him like on weekends, taking him to places if he needs something, shopping for him. Just being with him, keeping him company”. Another participant stated, “all of us see our mother every week. Most nights of the week for dinner, she has a child with her eating dinner with her. We make a big commitment to my mother.” (SP5 Sister). Although participants are happy to be able to provide care for their parents in the form of completing various care tasks, it is the opportunity to spend quality time with their parents that the participants tended to find most rewarding. “I just did some shopping for her here and there. And I take her to the hospital or things like that. But sitting down with her for an hour and talking with her probably gives me the most enjoyment.” (SP8 Brother).

The participants did their best to address the specific care needs of their parents. For example, “There’s not a lot of things that she still enjoys doing. She does love reading, so that’s something I try to always keep going for her. So I’m constantly bringing her new books or bringing her food...just trying to give her the best quality of life while she still has it.” (SP10 Sister). Other participants explored day programs that would also meet the needs of their parents. At times attempting to meet all of the parents care needs was challenging. One participant explained, “In fact, he (parent without dementia) often never asks us at all, we kind of have to snoop in, find out you know, on his calendar and plan accordingly. Often around him.” (SP9 Brother). Another participant also expressed difficulties, “It was a challenging time, but you have to do what you got to do to make sure that the family keeps going.” (SP10 Brother).

### Sisters often take the lead

The majority of sister and brother participants acknowledged that the sister assumed the role of care manager or organizer. Both siblings would often refer to sisters as “the boss” (SP7 Brother). Typically, this role meant that the sister would often take the lead in coordinating and delegating the tasks that needed to be completed for the parent with dementia. As one pair explained, with the sister reporting, “I’m more of the researcher and organizer of getting services done and looked at and connections with doctors and hospitals and veterans and (homecare services)” (SP6 Sister) and the brother reporting, “my sister has set up a google calendar and everything is on there and we will assign who does what” (SP6 Brother). Sister siblings indicated that it was a natural occurrence for them to assess the situation themselves, determine what needed to be done, and then delegate the tasks involved, based upon their personality. On the other hand, both sisters and brothers suggested that the brothers were very likely to step in and perform tasks when asked. Some examples of tasks the brothers would do included organizing appointments and transportation as well as hiring and delegating care provided by a personal support worker. Other common activities for brothers included management of parents’ finances and creating shopping lists for the other sibling(s). The care manager and organizer role of the sister was most commonly cited, regardless of the sex of the parent, the stage of dementia, or whether the parent had a spouse who also provided care.

Brother participants were described as taking a more passive role to providing care. For example, one participant expressed, “I just do as I am told (by my sister) and do what is needed when it is needed” (SP10 Brother) and another brother explained “My sister does more of the takes care of finances, delegate, like she may ask me to do something, or may send a group text out to say can you do this?”. The sisters would often take the lead and be proactive in organizing the care tasks and delegating who (brother, caregiver, spouse, etc.) would be able to complete the tasks depending on availability, efficiency, and circumstances.

The sibling pairs stated that sisters assumed the care manager role for different reasons. For example, one participant expressed “a lot of the responsibilities sort of falls to me without a conversation” (SP10 Sister), while another participant explained, “My sister is more involved in organizing care needs because of her connections at (institution) with the gerontologists” (SP7 Brother). Sisters appeared to more readily take on tasks, even if they made them feel uncomfortable because they understood the tasks needed to be done. Brothers may decline tasks that made them uncomfortable and believed their sisters would take care of these tasks.

“I do not like to bathe my parent. It’s uncomfortable. It’s personal. It’s easier for my sister to do it. She’s a woman so her bathing my mom as another woman seems to make more sense. It’s more comfortable.” (SP10 Brother)

## Practical issues affect sharing of caregiving activities

Participants reported several factors that affect how caregiving activities are shared among siblings. The most prominent factors include availability and proximity. All sibling pairs reported availability is the primary factor in how caregiving tasks are distributed. For example, “Our division is based on our time availability. I say that that’s number one, division” (SP9 Brother). Flexibility of work hours, scheduling, household demands, and lifestyle, including whether the participant had children or not, were the most commonly reported barriers influencing the amount of time a sibling could contribute to care. For example,

Most of it was based on availability, I was working two jobs, my sister was working and my wife had taken maternity so she was providing a lot of the interim care. And then we subbed off so that I could provide her assistance in the evening when mom was at her worst. (SP10 Brother).

Siblings who did not have children, have flexible work schedules, or were retired tended to contribute more caregiving hours as they were perceived to have more time. Furthermore, caregivers’ personal health challenges can also limit one’s availability as reported by one participant.

Most sibling pairs lived in the same city as their parent with dementia, but for two pairs, the sister lived several hours away. These sisters assume more administrative and coordinator roles. The brothers, who were closer in proximity, provided more physical help as required, including household maintenance or shopping. For example, a participant expressed, “I pretty much do most of the organizing and because I’m not physically in the same area geographically at the moment, I have to ask him (brother) to help with in-person chores.” (SP10 Sister). For other sibling pairs who all lived within the proximity of the parent with dementia, they tend to divide up the tasks based on availability. A participant explained “we’ve been in constant communication so we’re always talking about who’s where and which day works for which person and which things we’re struggling with” (SP9 Sister).

## Personal resources and/or skills affect division of responsibilities

Participants reported that individual skills and resources played a role in how caregiving tasks were divided. Caregivers discussed skills that they had because of work experience and/or their own personality as one participant explained,

All of us are university educated… I primarily say this is what my mother needs. This is my area of strength and I along with my brother who is the management consultant, manage almost entirely all the financials things. Making sure that people are paid, making sure the nursing home has what it needs. I do all the appointment scheduling… But for medical things, my two brothers (doctors) step in and responds to medical appointments. I would get my mother to the appointment, but my brothers would try to be available for that. We try to look at what our strengths are and we respond that way. (SP5 Sister).

If a specific task required a sibling’s expertise, they would be the ones responsible for that task. For example, sisters were more likely to perform in-person tasks (e.g., tangible assistance within the home), caregiving tasks that had immediate and noticeable outcomes (e.g., aid in dressing or bathing), while brothers were more likely to take on tasks that did not have to be completed in person and had more flexible time schedules (e.g., financial management). This could be partially explained by gender and how this influences career paths that males and females typically pursue. Sisters more often came from healthcare backgrounds, and consequently provided greater assistance with tasks related to self-care and hygiene. On the other hand, brothers were more likely to have greater experience regarding the financial aspects and property management elements of care. The sibling pairs reported that they found sharing care based upon expertise to be the most effective and fair way in distributing care tasks. It appeared that expertise helped create clear boundaries regarding the division of care tasks. These clear boundaries influenced how siblings communicated their caregiving roles with each other and to healthcare professionals.

Personality traits, including a person’s typical thoughts, feelings and behaviors, also played a role in how tasks are divided. Four sibling pairs alluded to how the sibling’s capacity determined the type of tasks that are divided. For example, one sister stated, “I also have capable and controlling tendencies to make sure that I get things done. I always get things done… that’s my nature and she (parent with dementia) depends on me.” (SP8 Sister). Siblings who were more organized tended to initiate conference calls, emails, and utilized web-based applications to help delegate tasks. Two sisters used calendar applications to organize and delegate tasks based on siblings’ availability and personal strengths. Siblings who had strengths in socialization skills tended to provide more emotional support and spent more quality time with their parent. For example, “I think that he is a good companion, he likes to sit and laugh with her. I don’t think we have a giant divide; I think it is more about the nature of my personality and my view of my skill set, that is more of the differentiation between the task we do.” (SP8 Sister).

Personal resources, such as help from their families, also affected task delegation. Five brothers reported that their wives were also involved in tasks such as cooking and attending appointments. The involvement of the person with dementia’s spouse, and/or private caregiver also affected how caregiving tasks were distributed amongst siblings. Three sibling pairs reported that their other parent was the primary caregiver and the participants ensured both parents’ needs were met. For example, “they’ve been very independent, and my mother actually does all the caregiving other than discussion and you know, sort of research that I do. But the actual physical care is still even being done by her. It’s more a support. A support done between my brother and I.” (SP6 Sister). Three sibling pairs reported that they hired private caregivers to provide assistance to their parent. Since the private caregivers tend to provide with self-care and cooking tasks, the sibling pairs focused on other aspects of their parent’s needs, such as shopping.

## Shared caregiving influences relationship quality

Sharing of caring responsibilities often contributed to positive or negative changes in the quality of the sibling relationship. Most of the participants reported that they had a good relationship with their sibling. Factors that influenced the quality of a sibling relationship included pre-existing relationship (e.g., having a good relationship prior to caregiving), communication (e.g., good and bad communication styles can have positive and negative consequences for relationships), and perceived equality in distribution of tasks (e.g., accepting that even though care was unequal, it could be equitable). Participants defined equitable caregiving as care tasks being divided fairly based upon each siblings’ circumstances.

Participants reported that their pre-existing relationship with their sibling affected the quality of their current relationship. As one participant shared, “we were never close, so it makes sense that we are still not close now given the extra stress” (SP13 Sister). Another participant shared that her brother and she were able to maintain the positive relationship they had before becoming caregivers: “We’ve always had good relationships. But I’m saying because our dad’s decline coincided with our mother’s loss, we became closer because we needed to in a way. So our circumstance, but also the more needy our father is, the more close we’ve become actually.” (SP9 Sister).

Positive communication between siblings tended to improve relationship quality. Many sibling pairs reported that their relationship remained the same or they have gotten closer since they started sharing caregiving tasks due to increased communication with each other and involvement in each other’s lives. As one participant explained, “As his dementia grows as he gets older and more needy, we need to dedicate more of our time together either discussing him or actually dealing with him. So that’s the change.” (SP9 Brother). However, some sibling pairs reported that poor communication impacted their perception of their relationship with their sibling. For example, one brother reported,

“Sometimes the boss (sister) would send out a text message that doesn’t sound very friendly. You know, I’m a salesman, so I know that a little bit of sugar goes a lot better than I mean… and my older sister especially would get pissed. You know, don’t talk to me this way, yada yada yada.” (SP8 Brother).

All of the sibling pairs reported that communication is key to preventing and resolving conflicts in their relationships. For example, scheduled communication with family was important to relieve stress that arose from caregiving tasks, “when my mother first came home from hospital, I just generated a massive list and started to get at it. Then I found myself overwhelmed, and my brothers were willing to help a bit, but what I have done is said we have to have conference calls.” (SP5 Sister). Another strategy was scheduled communication is to ensure all siblings were updated about their parent’s progress and care needs, “We don’t always completely agree but we actually have always tried to schedule regular calls because one of my brothers is always out of town, so we schedule time to connect, the three of us and discuss our ends” (SP9 Sister). In addition, open and honest communication about personal struggles with their sibling(s) allowed for more effective distribution of caregiving tasks. A participant explained, “people are willing to be honest with what pressures they have, like you know what, I’ve got a big issue at work, I can’t do this right now” (SP5 Sister).

Conflicts and resentment arose within sibling relationships due to perceived unequal distribution of caregiving tasks. As one participant explained,

“Do I have the level of intimacy that I would like with my sister? No, but I think that some of that has unfortunately been due to care because it is a very stressful situation and to be perfectly honest it was an unequal allocation of time and resources and to be fair, at that point … I would say we are not as close as we once were.” (SP10 Brother)

However, siblings described recognizing and appreciating their siblings’ efforts. This type of appreciation enhanced the quality of their relationship. For example, a brother stated that, “(My sister) was incredible on making sure that the process was driven forward even if I thought I did more, she did what she could.” (SP10 Brother). Furthermore, a sister was appreciative of her brother who took on more responsibilities, “I have a brother who generally has stepped up to the plate in a way that I would have never seen. It’s not equal but it’s what he can do. I’m thrilled” (SP8 Sister).

## Discussion and implications

This study helped to illuminate how mixed-gender sibling dyads shared caregiving responsibilities when caring for a parent with dementia, and the impact of shared caregiving on the quality of their relationship. Thematic analysis revealed five main themes: (1) the goal of shared caregiving is to meet parent needs, (2) sisters often take the lead, (3) practical issues affect sharing of caregiving activities, (4) personal resources or skills affect division of responsibilities, and (5) sharing care influences quality of sibling relationships.

Our study illustrated that even close sibling relationships were not impervious to conflicts and challenges within the relationship when tasks are not distributed evenly. This is particularly true when siblings perceived unequal distribution of caregiving tasks. Our findings are consistent with the research of Dwyer et al. (1992) and Ingersoll-Dayton et al. (2003), who found that sibling caregivers evolve into their caregiving roles through dynamic and regular practices of communication. Participants in our study described how they came into their caregiving roles through a process of communicating with their siblings, dividing tasks up, and sharing the common goal of meeting the needs of both the parent with dementia and, if available, their spousal caregiver. Participants described how this process evolved over time as sisters often took on the role of the organizer. Aronson (1992) found that adult children caregivers rarely plan for the division of care with their siblings. Although women in our study provided more care for their parents, few attributed this to their gender but rather to their goal of caring for their parent. Cultural constructions of gender can account for gendered caregiving patterns as many cultures endorse the value of woman as caregivers (Gubermanet al., 1992). However, as more men take on the caregiving role, gendered roles within families will evolve, influencing how care responsibilities are managed within teams (Robinson et al., 2014). By working as a team, conflict arising from misunderstandings may be reduced, since all siblings are working toward a common goal. Although most of our participants were able to work through their disagreements over time, caregivers can benefit from support and strategies to better communicate struggles and help caregivers navigate their new roles.

Feminist theories suggest that caregiving is often gendered with more women taking the lead caregiving role (Neysmith, 1995). Our study expands this discussion by highlighting the influence of expertise, personality, and comfort on the division of care tasks. Participants in our study described dividing tasks based upon the siblings’ expertise to be effective, resulting in sisters primarily taking on tangible tasks at home and brothers performing financial assistance. This mirrors the findings of others that also found siblings divide tasks based upon expertise (e.g., Connidis & Barnett, 2018; Ingersoll-Dayton et al., 2003a). Sisters described taking the lead in care and attributed this to their personality tendencies of liking to take control, get things done, and delegate to their brothers. This finding contradicts existing research with sons who reported taking on a care manager role and sisters providing emotional support (Grigorovich et al., 2016). Brothers in our study made contributions to non-functional tasks such as providing emotional support, financial support, and quality time, based upon their comfort with providing those tasks. In addition to expertise, personality, and comfort, the influence of gender on division of caregiving tasks may be also mediated by several other variables such as relationship, socioeconomic status (Bartlett et al., 2018; Kokorelias et al., 2021), situational factors (e.g., location and employment) (Kokorelias et al., 2020), gender (Xiong et al., 2020), and cultural background of the caregiver (Liu et al., 2021).

For brother–sister caregiving dyads, division of caregiving tasks is rarely equal, but it can be equitable. Equitable caregiving has been defined as the “distribution of caregiving tasks among siblings as being fairly shared in the context of facilitators or constraints” (Kokorelias et al., 2020, p. 3). Our study expands our understanding of how equitable caregiving can be achieved. It suggests care tasks are shared in the context of many different factors including expertise, personality, comfort, and practical issues. Participants in our study echoed previous research findings (Kokorelias et al., 2020) by suggesting equitable caregiving may improve the relationship quality between siblings caring for a parent with dementia. Healthcare professionals can facilitate quality relationships by helping siblings recognize one another’s strengths and practical issues that affect caregiving. Ultimately, siblings want to work together in their shared interest of caring for their parents and to perceive their caregiving situation as equitable. It is also important that healthcare professionals still offer support to caregivers who are perceived to be managing well (Lotherington et al., 2018), particularly female caregivers who may be at greater risk for depression and psychosomatic symptoms than male caregivers (Pillemer et al., 2018). Healthcare professionals can help caregiver siblings achieve equitable shared caregiving by helping them consider their strengths and practical issues that affect their ability to share care.

Most sibling pairs also reported good quality relationships with their siblings due to close pre-existing relationships and strategies to minimize or address conflicts and disagreements. Existing caregiving literature has predominating examined caregiver–care recipient relationships (e.g., Betts Adams et al., 2008; Li & Seltzer, 2003), rather than the relationship between caregivers. Consistent with previous findings, greater communication improved relationships between sibling caregivers (Matthews & Rosner, 1988; Ingersoll-Dayton et al., 2003b). Negative consequences were observed in siblings who contributed more time providing care and those who felt resentful towards their sibling counterparts (Ingersoll-Dayton et al., 2003b). The styles of participation as outlined by Matthews & Rosner (1988) were also observed in our participants. For example, siblings who worked together to develop a routine to providing care most often reported a good relationship. In poor sibling relationships, participants described that their siblings did not contribute as much time as they wished. Our study provides preliminary insight into the factors that influence relationship quality amongst caregiving sibling and suggests that it may be important that healthcare professionals encourage effective methods of communication between siblings to minimize conflicts. Future research may wish to consider the long-term impact of pre-existing sibling relationships on the caregiving experience over time.

## Study limitations

This study was an exploratory descriptive study with a homogenous sample of participants who were primarily white, English-speaking, highly educated, of high socioeconomic status. As a result, the findings may not be transferable to a more diverse population or reflect the experiences other caregivers providing care in areas with limited access to community services and supports. Caregivers included in the study voluntarily participated, and therefore, the results may be biased in regard to including siblings with a good relationship. As a result, this may only reflect situations where siblings have good communication and maintain close relationships. Siblings in a caregiving system that are dysfunctional, emotionally taxing, and conflicted may not agree to participate in research about sibling caregiving.

## Conclusion

This study adds to the limited available literature exploring how mixed-gender siblings negotiate caregiving tasks, and the quality of their relationship. We found that sisters often assume the role of case manager/organizer by taking the lead in determining and delegating care tasks and needs to the rest of siblings or brother. Second, caregivers’ personal resources, skills, and knowledge influence delegation of tasks as it is common to play on the siblings’ areas of strength. Last, siblings have identified that open, honest communication is key to understanding each other’s struggles to better address inequalities in providing care for a parent with dementia and dissatisfactions in their relationship. This research informs the provision of support interventions for siblings caring for a parent with dementia. Healthcare providers can promote positive experiences in care division and conflict navigation for sibling networks caring for a parent with dementia by coaching open communication and promoting division of labor based on practical considerations and individual strengths. Longitudinal studies would provide insight into the needs and experiences of sibling caregivers across the dementia caregiving trajectory. Additionally, further research is needed to understand other variables that can account for gender differences among mixed-gender sibling pairs. As more siblings provide care to a parent with dementia, it will become ever more critical to best understand how siblings share caregiving responsibilities to help support siblings in their caregiving roles.

## Supplemental Material

sj-pdf-1-dem-10.1177_14713012211053970 – Supplemental Material for Brothers and sisters sharing in the care of a parent with dementia Click here for additional data file. Supplemental Material, sj-pdf-1-dem-10.1177_14713012211053970 for Brothers and sisters sharing in the care of a parent with dementia by Kristina M Kokorelias, Nira Rittenberg, Amy Law, Natasha T Chin Wan, Jennifer Machon, Yasmin Arfeen and Jill I Cameron in Dementia
