# Supplementary material for: Brothers and sisters sharing in the care of a parent with dementia
Source: Dementia (London). 2022 Feb 3;21(3):765–80. doi: 10.1177/14713012211053970 (PMC8996302; doi:10.1177/14713012211053970)
Supplement: sj-pdf-1-dem-10.1177_14713012211053970 – Supplemental Material for Brothers and sisters sharing in the care of a parent with dementia [file sj-pdf-1-dem-10.1177_14713012211053970.pdf]

Supplemental Material

Appendix 1: Sample Interview Questions

Interview topics include:

(1) Quality of relationship with siblings.

a) Describe your relationship with your sibling before entering your role as a caregiver.

b) Describe your current relationship with your sibling.

(2) How caregiving roles are negotiated.

a) Can you describe how you and your sibling(s) decide who would perform certain caregiving tasks?

b) If you wanted to change your caregiving roles (add or remove), how would you do this?

(3) Type of care provided.

a) Can you describe how you and your sibling(s) decide who would perform certain caregiving tasks?

b) How might your gender influence the caregiving tasks you perform for your parent?

Commented [KK1]: Supplementary material can be made available by the publisher online only and linked to the published article. This material includes supporting material that is not essential for inclusion in the full text to understand the conclusions of the paper but contains data that is additional or complementary and directly relevant to the article content and therefore may benefit the reader. Such information might include more detailed methods, extended data sets/data analysis, or additional figures.
It is standard practice for appendices to be made available online only as supplementary material. All text and figures must be provided in suitable electronic formats. All material to be considered as supplementary material must be submitted for peer review at the same time as the manuscript and included in the anonymous and nonanonymous versions of the manuscript (do not upload the material as separate files) and indicated clearly as supplementary material. Also ensure that the supplementary material is cited in the main manuscript where necessary, for example, "(see Supplementary Material)' or "(see Supplementary Figure 1)." The material cannot be altered or replaced after the paper has been accepted for publication, and **it will not be edited**.
Appendices
All appendices will be published online only as supplementary material (please see FORMATTING and SUPPLEMENTARY MATERIAL instructions above).

For Peer Review
